# Cancer stem cells – the current status of an old concept: literature review and clinical approaches

**DOI:** 10.1186/0717-6287-47-66

**Published:** 2014-12-10

**Authors:** Lukasz Fulawka, Piotr Donizy, Agnieszka Halon

**Affiliations:** Department of Pathomorphology and Oncological Cytology, Wroclaw Medical University, ul. Borowska 213, 50-556 Wroclaw, Poland; Department of Pathomorphology, Lower Silesian Oncology Centre, pl. Hirszfelda 12, 53-413 Wroclaw, Poland

**Keywords:** Cancer stem cells, Stem cells, Tumour-initiating cells, Tumour-propagating cells, Carcinogenesis, Tumour heterogeneity, Clonal evolution

## Abstract

As regards their morphology and biology, tumours consist of heterogeneous cell populations. The cancer stem cell (CSC) hypothesis assumes that a tumour is hierarchically organized and not all of the cells are equally capable of generating descendants, similarly to normal tissue. The only cells being able to self-renew and produce a heterogeneous tumour cell population are cancer stem cells. CSCs probably derive from normal stem cells, although progenitor cells may be taken into consideration as the source of cancer stem cells. CSCs reside in the niche defined as the microenvironment formed by stromal cells, vasculature and extracellular matrix. The CSC assays include FACS sorting, xenotransplantation to immunodeficient mice (SCID), incubation with Hoechst 33342 dye, cell culture in non-adherent conditions, cell culture with bromodeoxyuridine. CSCs have certain properties that make them resistant to anticancer therapy, which suggests they may be the target for potential therapeutic strategies.

## Introduction

The concept of cancer stem cells (CSCs) has attracted researchers’ attention since the beginning of the 21^st^ century. It is noteworthy that this year marks the 20^th^ anniversary of the first experimental proof of CSCs existence [1]. Tumour cells are heterogeneous in terms of morphology, metabolism, proliferation rate, ability to metastasise and other features. Cancer stem cell hypothesis assumes hierarchical cellular structure of a tumour, analogous to normal tissue. The three basic functional groups of cells are stem cells, progenitor cells and mature cells [2]. Stem cells are a minor population. They are able to self-renew and differentiate towards mature cells [3, 4]. Stem cells rarely divide to give descendant stem cells or progenitor cells. The latter (also known as progenitors or transit-amplifying cells) proliferate intensively. Their descendants have a more restricted potential and are able to differentiate towards a certain type of mature cells. Progenitors have reduced capacity of self-renewal with a limited number of divisions, in contrast to stem cells which can divide throughout the lifespan of the organism [4]. Mature cells are the last stage of cellular development. Having lost the ability to divide, they contribute to the role of the tissue which they form.

Normal tissue is characterized by a fixed number of cells. Dying mature cells are replaced by new-born mature cells derived from progenitors. This process is strictly controlled by mutual interactions between every cell forming the tissue. The delicate equilibrium is disturbed in carcinogenesis. Cancer progenitor proliferation gets out of control and the number of cells increases, which is one of the tumour defining features.

The aim of this paper is to introduce and briefly describe cancer stem cell concept. We are aware of the fact that exhaustive review of this subject is impossible within the confines of one work. Additionally, the current opinions about the role of CSCs in generating tumour heterogeneity and their potential clinical implications have been presented in this paper.

### Historical review

The “stem cell” term was first used by a Russian researcher Alexander A. Maximow as early as 1909 [5]. The era of intensive research on stem cells began in the mid-20^th^ century. In the 1950s Makino et al. showed in the series of experiments that cancer cell population isolated from peritoneal fluid of rats contains a certain subpopulation characterized by a specific karyotype. It was proved that these cells were present in every serially grafted derivative tumour [6, 7].

In the 1960s Pierce et al. published the results of their research, during which they isolated cells from embryonal bodies of teratocarcinoma (the term was used to describe a mixed type of tumour composed of teratoma and embryonal carcinoma but has been largely abandoned now) [8]. The cells were capable of differentiating into mature tissues [2]. Later Pierce and Speers coined the hypothesis that tumours were “caricatures” of normal tissues [2, 9].

In 1961 Till and McCulloch grafted hematopoietic cells from bone marrow of a healthy mouse into a host-mouse whose bone marrow had been destroyed by ionizing radiation. They proved that these cells gave rise to islets of hematopoietic stem cells in the spleen, which differentiated towards mature blood cells [2, 10, 11]. Thus, the two basic features defining stem cells, namely self-renewal and ability to differentiate into mature cells, were revealed. In 1977 Hamburger and Salmon observed a minor population of cells with the characteristics of stem cells in certain types of tumours [12].

The new era of research into CSCs started in the 1990s when their presence was proved experimentally. In 1994 Lapidot et al. reported on their breakthrough experiment. They showed that the CD34^+^/CD38^-^ cells population (phenotype characteristic for hematopoietic stem cells) of acute myeloid leukaemia (AML) is able to form derivative leukaemia after transplantation into NOD/SCID (non-obese diabetic/severe combined immunodeficient) mice [1]. It must be also stressed that populations of a different immunophenotype did not have this ability.

Since then serial cell transplantation into NOD/SCID mice has been used as a gold standard in CSC research [13], as it fulfils two crucial criteria defining CSCs - self-renewal and ability to form heterogeneous tumour cell population.

However, some researchers are sceptical about CSC hypothesis. They claim that the results of research on NOD/SCID mice xenotransplantation model are not sufficient to prove the existence of CSCs. There can be other reasons why a certain cell population is capable of generating a secondary tumour after grafting, while another one is not. The host’s microenvironment is a likely cause. In fact, immunity in NOD/SCID mice still exists and is mediated largely by NK cells [14, 15]. Moreover, research showed that cells of non-CSCs phenotype can form a secondary tumour after transplantation into congenic mouse (congenic - differing in one locus of the genome) [16, 17].

### Definition of CSCs

Due to a growing interest in CSCs, a Workshop on Cancer Stem Cells was convened in 2006 by the American Association for Cancer Research (AACR). The definition of a cancer stem cell that was arrived at in the Workshop has been generally used since that time. CSC was defined as “a cell within a tumour that possesses the capacity to self-renew and to cause the heterogeneous lineages of cancer cells that comprise the tumour” [13].

**Figure 1:**
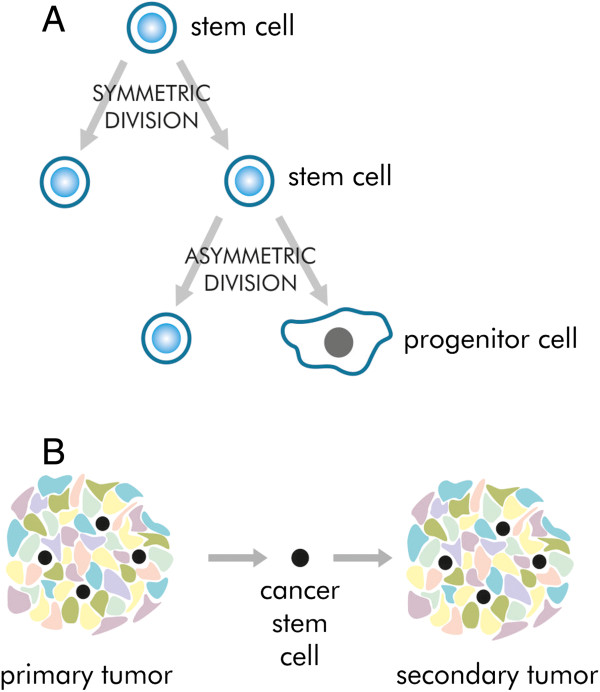
**Two features defining cancer stem cells. A** – Self-renewal: the ability to generate descendants retaining stemness characteristics. Stem cells undergo symmetric division to give two stem cells or asymmetric division, where one descendant remains a stem cell, whereas the other cell loses stemness features. **B** - Restoration of the heterogeneous cancer cell population. The secondary tumour is composed of the same cell types as the primary tumour.

What does the term “self-renewal” mean? Every tissue is a dynamic structure composed of cells characterized by a given lifespan which is generally much shorter than the lifespan of the whole organism. In this respect brand new cells need to be produced to substitute for the dying ones. They are generated from resting cells (i.e. cells with low biochemical activity and rarely dividing) defined as stem cells [2]. Their number needs to be constant to maintain the tissue alive. To achieve this goal, at least one cell needs to be a copy of its mother cell (Figure 1A). When the other cell is directed into a differentiation program, the division is defined as asymmetric [2, 7, 18]. The other mechanism of stem cells division produces two identical stem cells and is referred to as a symmetric cell division. Therefore self-renewal is defined as the ability to generate descendants retaining stemness characteristics [7].

The ability to produce a heterogeneous cell population is linked to cells isolated from a tumour (Figure 1B). To show this feature the experimental model should be used. The most appropriate model is transplantation into an immunosuppressed organism whose immune system does not reject grafted cells. The above mentioned NOD/SCID mouse model is generally accepted in this issue [13]. The ability to generate a heterogeneous secondary tumour cell population, which is identical to the primary tumour, denotes that there were CSCs amongst transplanted cells. The term “tumour-initiating cell” or “tumorigenic cell” are often used to emphasize this feature and thus can be treated as the synonyms for CSC. However, these terms can lead to a confusion with the first cell that was initiated and gave rise to cancer in the patient [13, 19]. For that reason some authors avoid using these phrases and propose the term “tumour-propagating cells” (TPCs) [19].

### Tumour heterogeneity

Morphological diversity of cells, as seen under a microscope, is much more pronounced in the tumour compared to normal tissue. It is one of the features of atypia, the term used to define malignancy in histopathology. There are also differences amongst tumour cells in a phenotype (for instance: expression of surface antigens and cytoplasmic proteins, activity of biochemical processes) and functionality (for instance: proliferation rate, invasion, metastases forming, activation of neoangiogenesis, resistance to systemic therapy) [3, 20]. The key factors responsible for tumour heterogeneity are genomic heterogeneity, hierarchical organization of tumour tissue, environmental influences and random processes [21, 22].

**Figure 2:**
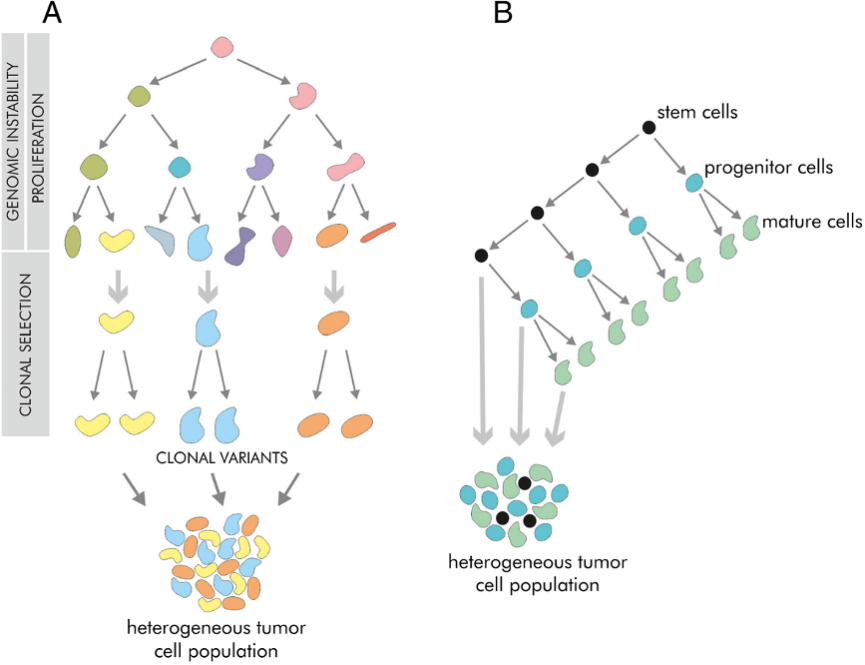
**Basic tumour heterogeneity models. A** - Clonal evolution model. High proliferation and genomic instability result in a large number of cells differing in genotype and thus phenotype. The best fitted cells are selected by Darwinian processes to generate clonal variants of the tumour. **B** - Cancer stem cell model. CSC population is capable of unlimited number of divisions. Tumour heterogeneity results from existence of phenotypically diverse populations of different stages of cell maturation.

Genomic heterogeneity results from genomic instability and increased proliferation rate [20, 21]. Mutated cells undergo natural selection in the Darwinian evolution mechanisms (Figure 2A) which favour better adjusted cells. These cells live longer and give rise to descendant cells. The clones are generated as tumour grows. Thus tumour mass is heterogeneous as it consists of clonal variants [21, 22].

The cancer stem cell model assumes that tumour tissue is hierarchically organized. CSCs population is responsible for tumour growth and progression (Figure 2B). In this respect heterogeneity means presence of cells at different stages of maturation [21].

**Figure 3:**
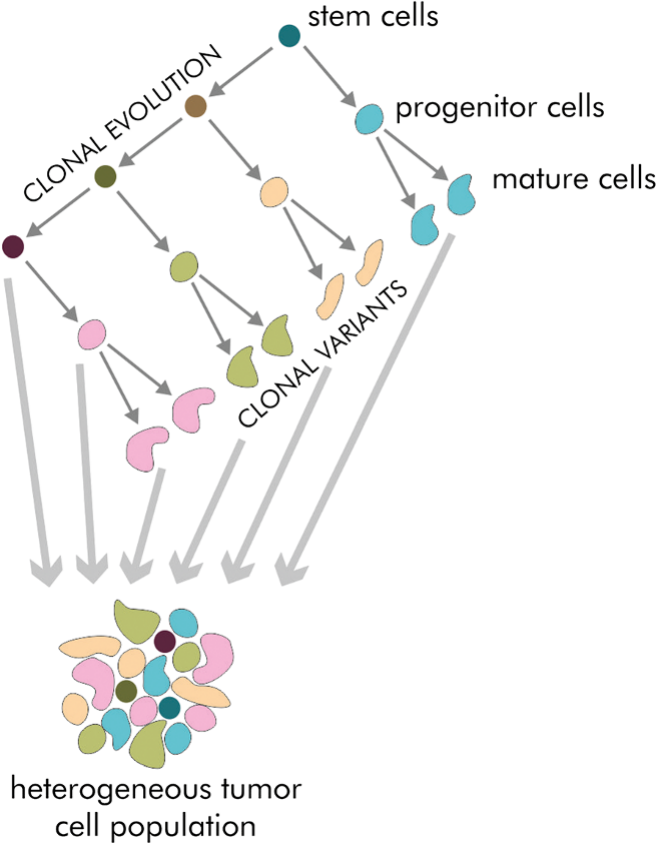
**Clonal evolution and CSCs model are not exclusive.** The population of CSCs may undergo clonal evolution. Tumour heterogeneity results from existence of both clonal variants and different stages of cell maturation.

Clonal evolution and CSC models describe the basic mechanisms leading to tumour heterogeneity [21]. Genomic heterogeneity has been proved by genomic research results [23]. The question that remains is whether most of tumour cells or only CSCs undergo clonal evolution. It is possible that only a minority of tumours are hierarchically organized and clonal evolution of CSCs occurs only in these cases [24] (Figure 3).

Like normal tissue, tumour cells are prone to influences from the microenvironment (stromal cells, extracellular matrix). The difference is that tumour tissue is characterized by a profound disarrangement of microenvironment. A wide variety of microenvironmental influences contributes to tumour cell heterogeneity [21]. The random (stochastic) processes result from random biochemical reactions. There is also another phenomenon referred to as transcriptional noise. It works by difference in the time of transcription between cells [21].

### The sources of CSCs

**Figure 4:**
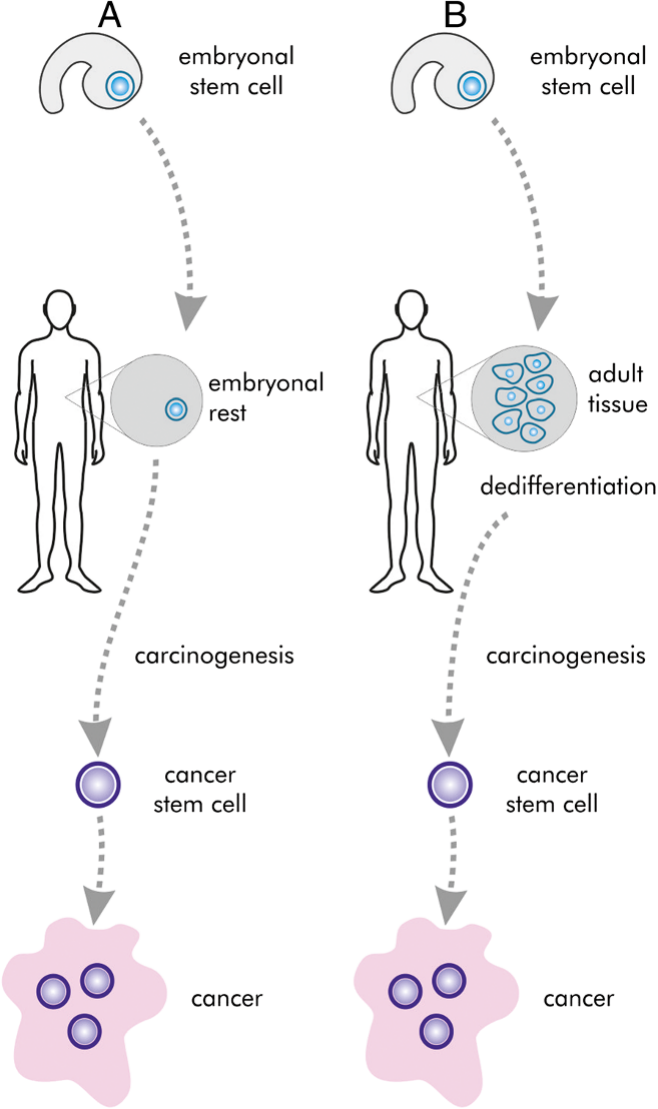
**The historical concepts of CSCs origin. A** - Embryonal rest theory. The pluripotent embryonal cells remain in the adult organism in the form of “embryonal rest”. They are the origin of CSCs. **B** - Dedifferentiation theory. Somatic stem cells of adult tissue gain pluripotency through dedifferentiation.

The concept of CSCs has been discussed in the scientific literature since the 19^th^ century. In 1874 Durante hypothesised that tumours derive from a rare cell population of stem cell characteristics [7]. Simultaneously, Conheim (Virchow’s student) speculated that these cells may be embryonal cells, which remain in the adult organism, retaining their pluripotency (Figure 4A). This concept was called the “embryonal rest theory” [2, 7]. In the late 19^th^ century this hypothesis was gradually replaced by dedifferentiation theory of carcinogenesis (Figure 4B). It assumed that adult differentiated cells are the source of cancer stem cells after process of dedifferentiation, i.e. reversal of differentiation [2]. In the mid-20^th^ century, when stem cells were gaining more attention, the concept binding together tumours and stem cells became attractive again.

It is a common mistake to treat a CSC as a synonym of a normal stem cell which has gone through carcinogenesis to initiate tumour [13, 24]. In this regard, some authors prefer using the term “tumour-initiating cells” [25]. To describe the former entity, the term “cancerous stem cell” can be used [26]. There are discrepancies between investigators regarding the source of CSCs. Intuitively, normal stem cells are likely to be the target of oncogenic initiation leading to the formation of CSCs [13, 26].

Similarly to normal tissue, CSCs give rise to progenitor cells which are an intensively proliferating cell population. Normal progenitors differentiate after a certain numbers of divisions and lose their ability to divide (terminal differentiation). However, cancer progenitor cells’ ability to proliferate is much higher. Thus, their progeny is much more numerous and accumulates, which leads to an increase in tumour mass [2].

CSCs may also derive from normal progenitor cells which acquired self-renewal ability in the course of accumulating mutations during carcinogenesis [13, 25]. Some experiments seem to confirm this hypothesis. In one of them, acute myeloid leukaemia was generated after transplantation of hematopoietic progenitor cells with transduced MLL oncogene [18, 27]. The results of the last research revealed unexpectedly that CSCs may derive from differentiated epithelial cells in the process of epithelial-mesenchymal transition (EMT) [28, 29].

### Epithelial-mesenchymal transition and metastases

Epithelial-mesenchymal transition is a process occuring during development of multicellular organisms. The epithelial cells acquire mesenchymal properties by loss of cell-cell junctions and polarity [29]. Owing to migratory and invasive capabilities, mesenchymal cells are concordant with cancer phenotype. Thus it has been proposed that EMT contribute to cancer development. The results of recent studies suggest that EMT produces cells with CSCs features in breast, pancreatic and colorectal cancers [30].

The metastasis process begins with detachment of cells from primary tumour and migration into the lumen of blood or lymphatic vessels (intravasation). The cells gain these features during EMT. The concept that CSCs may be metastatic precursor is supported by the fact that expression of CSCs markers by tumour seems to predict metastases [25]. The crosstalk between CSCs and EMT is also confirmed by the coexpression of stemness and mesenchymal-like profile in epithelial tumours [28]. The Wnt pathway seems to be the clearest molecular connection between EMT and stemness [28].

### CSC niche

Stem cells reside in the niche which is defined as a microenvironment made up of adjacent stromal cells, vessels and extracellular matrix [18]. The cells are sustained in undifferentiated state by the niche, which protects them from factors stimulating differentiation. The other way to sustain stemness by the niche is to limit the proliferation rate of stem cells [13, 31]. The elements forming the niche adhere to stem cells with adhesion molecules and control their function by signalling molecules, such as Shh (Sonic hedgehog), BMPs (bone morphogenic proteins) and Notch [32]. The constant number of stem cells may be also maintained by limited physical space of the niche. If cell division occurs in the “completely occupied” niche, one of the descendant cells must leave the niche. It then begins to differentiate because it is not exposed to niche factors maintaining stemness. This process is called asymmetric cell division [31, 32] that was referred to above. On the contrary, if there is free space in the niche, two descendant cells stay in it and are sustained in stemness [31]. This phenomenon is referred to as a symmetric cell division.

CSCs, similarly to their healthy counterparts, retain their self-renewal ability by interaction with the niche [13]. As the tumour grows, the number of niche cells probably increases. This phenomenon may be due to the stimulation of the niche cells to proliferate by CSCs [33]. Niche elements may be also transformed to lose their ability to control proliferation of themselves and of stem cells [33]. The transformation may also modify the niche cells to produce stimulating proliferation factors [13]. It may drive clonal selection of mutated stem cells and transform them into CSCs. It is also possible that stem cells gain independence from factors suppressing their proliferation (including the niche) or get the ability to occupy other niches on their way through carcinogenesis [13, 33].

### Methods of detection and isolation of CSCs

**Figure 5:**
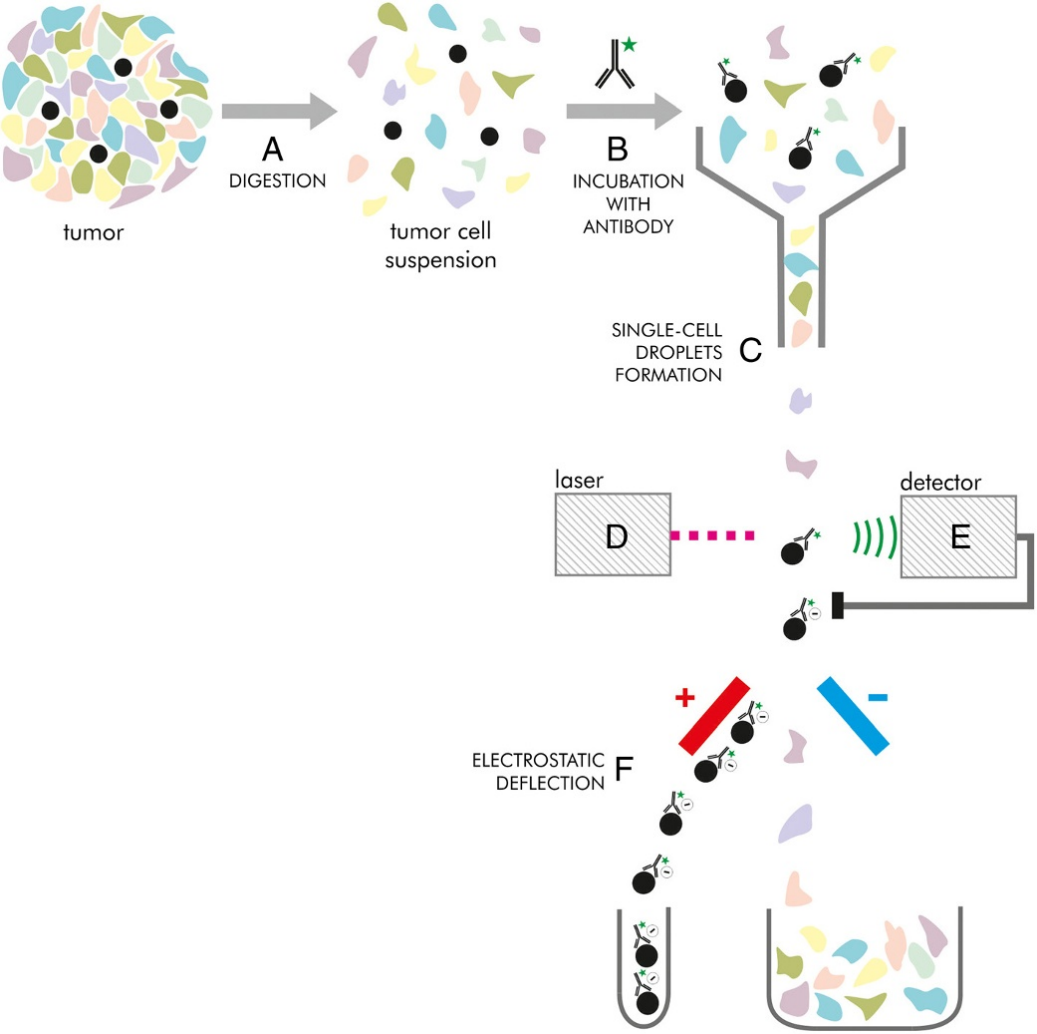
**The stages of CSCs isolation by FACS. A** - Solid tumour is digested by enzymes to cell suspension. **B** - Tumour cell suspension is incubated with antibodies directed against antigens specific for CSCs conjugated with fluorescent dye. **C** - The opsonized cell suspension is let through a narrow tunnel to form a single-cell-diameter stream. The vibration produces droplets containing single cells at the mouth of the tunnel. **D** - The droplets pass through a laser beam. The fluorescent dye is excited to emit the light identified by a detector. **E** - The detector is paired with the device which gives electrostatic charge to light-emitting cells. The “dark” cells remain uncharged. **F** - The flow of electrostatically charged cells is bent by the electric field. The cells are collected in the tube. The uncharged cells fall by gravitation.

According to the definition coined by AACR Workshop on Cancer Stem Cells, tumour cell population could be defined as CSCs if it is experimentally showed to produce a secondary tumour which is composed of identical heterogeneous cell population as the primary tumour [13]. To prove it, the cell population which is examined, needs to be isolated from other cells forming the tumour. A specialized type of flow cytometry, FACS (fluorescence-activated cell sorting) is used (Figure 5) for that purpose. If a solid tumour is examined, it is exposed to enzymes that degrade intercellular junctions and bounds between the cells and extracellular matrix. Then, the cell suspension flows through a narrow tunnel to form a single-cell stream. It is directed into a vibrating nozzle which breaks it apart into droplets containing single cells [34]. Every droplet goes through a laser beam. Cells differ in their optical characteristics, i.e. rate of absorption, emission and dispersion. These features are caught by detectors. Then, cells of certain characteristics are charged electrostatically and they are directed to electrostatic field. The phenomenon of electrostatic deflection bends the charged cell flow. The cells are collected in the vessel [34]. The remaining electrostatically uncharged cells flow vertically down.

FACS can be used to isolate cells differing in surface markers phenotype [34]. In this case, cell suspension is incubated with an antibody specific for a certain surface antigen, conjugated to fluorescent dye. Then cells flow through a laser light beam of wavelength adjusted to a fluorescent dye used. Opsonized (thus expressing certain antigen) cells are isolated using electrostatic deflection described above.

Alternatively, antibody bound to magnetic beads can be used. In this method, called magnetic-activated cell sorting (MACS), the cell suspension stream into a strong magnetic field. The cells expressing marker specific to the antibody used, stay in the magnetic column, while other cells flow through. Later, the column is removed from the magnetic field and the remaining cells are washed out.

Another distinctive feature of CSCs is their ability to actively move xenobiotics out of them. It is defined as efflux. It results from increased expression of membrane proteins of ABC family. These proteins are responsible for Hoechst 33342 dye efflux [7, 35]. Cell suspension is incubated with Hoechst 33342 and then FACS sorting is used. The dye-negative fraction is called side population (SP) [7, 36]. However, it is generally assumed that SP population is not synonymous with CSCs. Some researchers hypothesise that it may even not contain CSCs [13].

**Figure 6:**
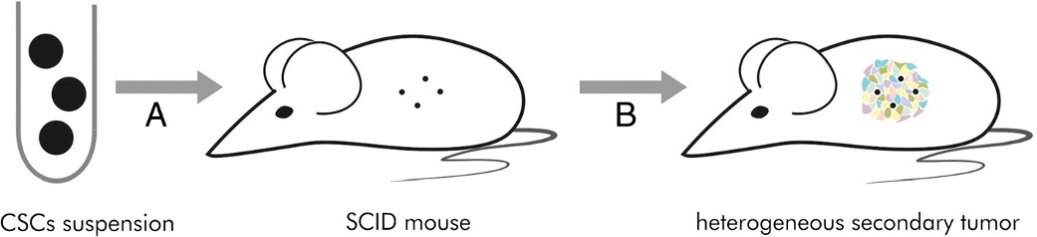
**CSCs xenotransplantation into SCID mouse. A** - Cell suspension containing FACS-isolated CSCs is injected subcutaneously or intraperitoneally into SCID mouse. **B** - Generation of a secondary tumour with heterogeneous population, analogous to the primary tumour, is highly conclusive of CSCs existence in cell suspension.

The gold standard in CSC research, as mentioned above, is xenotransplantation into immunodeficient animals. To examine if a certain cell population contains CSCs using this method, the candidate cells need to be isolated first. FACS sorting is used for this purpose. Then, isolated cells are injected subcutaneously or intraperitoneally into mice (Figure 6). When a secondary tumour is formed in a host organism, the procedure of isolation and transplantation is repeated. If it gives rise to a tertiary tumour consisting of identical heterogeneous cell population as the primary tumour, it is highly probable that the examined cells are CSCs [13].

Another method to detect CSCs is adhesion free cell culture. Every cell remains in suspension for its entire lifespan. If a certain cell gives rise to the population of progeny, they stay bound together in the form of a free floating colony of spheroid shape [7].

CSCs, similarly to normal stem cells, proliferate rarely. This feature is detected by label retention assay [13]. The most commonly used one is bromodeoxyuridine (BrdU). This nucleotide is incorporated into the DNA. The more cell divisions occur, the more diluted BrdU becomes. Thus CSCs retain more BrdU than other cells.

### CSC markers

There are no universal markers of CSCs. In addition, no potential marker is uniquely specific for stem cells. It is a common mistake to assume that the phenotype of CSCs of a certain tumour could be identical or even similar in the other type of tumour [13]. Amongst many potential markers of CSCs, two most numerous groups can be highlighted, i.e. membrane antigens and transcription factors. Only well-established markers are discussed here since we assumed that it was impossible to describe all of the potential CSCs markers in this paper.

The first malignancy proved to contain CSCs was acute myeloid leukaemia. It was shown that leukemic stem cells possess CD34^+^CD38^-^ phenotype [1]. Breast cancer was the first solid tumour that CSCs were isolated from. It was proved that a relevantly lower number of CD44^+^/CD24^-^ cells was able to initiate a secondary tumour after grafting into NOD/SCID mouse than any other phenotype [37]. The expression of CD44 antigen was then revealed in cells initiating prostate cancer [38], pancreatic cancer [39] and head and neck squamous cell carcinoma [40]. The other surface antigen, CD133, was proved to be a marker for stem cells of brain tumours [41], colorectal cancer [42, 43] and lung cancer [44]. CD90^+^/CD45^-^ phenotype was revealed in CSCs of hepatocellular carcinoma [45]. For practical purposes, surface markers are the most useful, due to the fact that they allow the isolation of intact cells.

In 2006 Takahashi and Yamanaka described their experiment during which different transcription factors were introduced into mouse fibroblasts [46]. They proved that only four of them (Oct4, Sox2, c-Myc and Klf4) were sufficient to gain pluripotency (ability to differentiate into every tissue of the organism). These cells were named induced pluripotent stem cells (iPSCs) and the four factors were later called Yamanaka’s factors [8]. Soon after that Yu et al. generated iPSCs from human somatic cells [47]. In this case only three factors (Oct4, Sox2 and Nanog) were sufficient to create iPSCs. The expression of these factors was revealed in prostate cancer stem cells [48]. Oct4 marker was also proved to be CSCs marker of breast [49] and urinary bladder cancer [50]. For the abovementioned reason, the transcription factors are less useful in functional testing than surface markers.

ALDH (aldehyde dehydrogenase) is considered to be yet another important marker of CSCs [7]. Breast cancer was the first tumour whose stem cells were showed to have increased ALDH1 isoform activity [51]. The elevated activity of this enzyme was also revealed in acute myeloid leukaemia [52], prostate cancer [53] and hepatocellular carcinoma [54].

It is worth to mention that a subset of cells isolated on the basis of certain markers expression is not equivalent of CSCs population. If it were true, every single isolated cell would be capable of spheroid formation or generation of secondary tumour after xenotransplantation. As we know, more than one cell is needed to accomplish it. Moreover, the definition of CSCs in some tumours has been narrowed after additional research. The further refinements of CSCs phenotypes are expected, as it happened recently in the case of breast cancer. In this example, the initial definition of CD44highCD24low cells was narrowed to a subset additionally expressing ganglioside GD2 [55].

Furthermore, distinct subsets within the same tumor entity can show stemness characteristics. A good example is glioblastoma, where both CD133^+^ and CD133^-^ subtypes were similarly tumorigenic in nude mice in vivo [56]. It was shown that these subsets were characteristic for different tumour subtypes (mesenchymal and pro-neural) - which had not been appreciated on histological examination [56]. In this respect, we cannot expect clearly defined CSCs markers to be specific for certain tumour entity. On the other hand, expression of CSCs markers can enable to divide certain diagnostic entity into prognostic group. For example, research revealed that CD133 expression in oligodendroglial tumors indicated shorter survival and predicted poorer clinical outcome [57].

### The role of CSCs in anticancer therapy

Classical anticancer strategies (chemotherapy and radiotherapy) kill intensively proliferating cells, which leads to cytoreduction and regression of malignant lesion. The cancer stem cell hypothesis assumes that CSCs are the source of every cancer cell. They are a rarely dividing population, so anticancer agents may not eradicate them, which may lead to the development of minimal residual disease (MRD), which in turn may be the cause of recurrence [14, 18, 58].

Moreover, CSCs have inherited or acquired resistance to anticancer therapy. The reasons for that may be elevated activity of mechanisms of DNA damage detection and repair, aberrations in apoptotic pathways, increased ability of xenobiotic efflux, reduced production of free radicals or elevated production of certain interleukins [14, 58, 59].

Efficient anticancer therapy should eliminate cancer stem cells as the potential source of recurrence. In this respect, CSCs are a promising target for potential therapeutic strategies. It must be emphasised that drugs must be as specific as possible towards CSCs to spare healthy stem cells.

There are a few potential therapeutic strategies against CSCs: direct killing by a chemotherapeutic agent bound to an antibody specific for membrane antigen, suppression of self-renewal pathways, blocking of therapy-resistance mechanisms and induction of differentiation [14]. However, it is yet too early to say if all or any of these methods would result in victory in the battle against cancer.

## Conclusions

The concept of crosstalk between stem cells and cancer appeared as early as in the 19th century. The existence of CSCs was experimentally proved in 1990s. Currently, the cancer stem cells hypothesis assumes hierarchical cellular structure of a tumour, with CSCs population capable of self-renewal and production of a heterogeneous tumour cell population. The number of potential CSCs markers have been recently reported. The ongoing research reveals the possible role of CSCs markers as a prognostic factors in cancer diagnostics. Moreover, they can act as the target for future anticancer therapy.

## Abbreviations

AACR: American Association for Cancer Research
ABC: ATP-binding cassette
ALDH: Aldehyde dehydrogenase
AML: Acute myeloid leukaemia
BMPs: Bone morphogenic proteins
CSCs: Cancer stem cells
EMT: Epithelial-mesenchymal transition
FACS: Fluorescence-activated cell sorting
iPSCs: Induced pluripotent stem cells
MACS: Magnetic-activated cell sorting
MRD: Minimal residual disease
NOD/SCID: Non-obese diabetic/severe combined immunodeficiency
Oct4: Octamer-binding transcription factor 4
SP: Side population.
